# Prediction of glycaemic control in young children and adolescents with type 1 diabetes mellitus using mixed-effects logistic regression modelling

**DOI:** 10.1371/journal.pone.0182181

**Published:** 2017-08-02

**Authors:** Michiel Joost van Esdonk, Bonnie Tai, Andrew Cotterill, Bruce Charles, Stefanie Hennig

**Affiliations:** 1 School of Pharmacy, Pharmacy Australia Centre of Excellence (PACE), The University of Queensland, Woolloongabba, Queensland, Australia; 2 Pharmacy Department, The Prince Charles Hospital, Chermside, Queensland, Australia; 3 Lady Cilento Children’s Hospital, South Brisbane and Queensland Paediatric Endocrinology, Woolloongabba, Queensland, Australia; Universitatsmedizin Greifswald, GERMANY

## Abstract

**Objectives:**

Glycaemic control in children and adolescents with type 1 diabetes mellitus can be challenging, complex and influenced by many factors. This study aimed to identify patient characteristics that were predictive of satisfactory glycaemic control in the paediatric population using a logistic regression mixed-effects (population) modelling approach.

**Methods:**

The data were obtained from 288 patients aged between 1 and 22 years old recorded retrospectively over 3 years (1852 HbA1c observations). HbA1c status was categorised as ‘satisfactory’ or ‘unsatisfactory’ glycaemic control, using an *a priori* cut-off value of HbA1c ≥ 9% (75 mmol/mol), as used routinely by the hospital’s endocrine paediatricians. Patients’ characteristics were tested as covariates in the model as potential predictors of glycaemic control.

**Results:**

There were three patient characteristics identified as having a significant influence on glycaemic control: HbA1c measurement at the beginning of the observation period (Odds Ratio (OR) = 0.30 per 1% HbA1c increase, 95% confidence interval (CI) = 0.20–0.41); Age (OR = 0.88 per year increase, 95% CI = 0.80–0.94), and fractional disease duration (disease duration/age, OR = 0.80 per 0.10 increase, 95% CI = 0.66–0.93) were collectively identified as factors contributing significantly to lower the probability of satisfactory glycaemic control.

**Conclusions:**

The study outcomes may prove useful for identifying paediatric patients at risk of having unsatisfactory glycaemic control, and who could require more extensive monitoring, support, or targeted interventions.

## Introduction

Appropriate glycaemic control in children and adolescents with type 1 diabetes mellitus (T1DM) is crucial for reducing potentially serious adverse effects such as ketoacidosis, nephropathy, microangiopathy, neuropathy and diabetic retinopathy in later stages of the disease [1]. Targeted glycaemic control to lower blood glucose levels has been shown to reduce the risk of developing multiple complications [2,3]. Therefore, the identification of patients at risk of poor glycaemic control before the onset of these complications is important. The management of T1DM can involve complex interactions between diet, lifestyle, medication compliance, management of comorbidities, and the input of carers (e.g. parent, guardian) and healthcare professionals (e.g. diabetes educator, dietitian, psychologist) in attempting to reduce high blood glucose concentrations towards ‘normal’ values [4,5]. The most common means of monitoring glycaemic control involves glycosylated haemoglobin A_1c_ (HbA1c) measurements, which provides a retrospective ‘longitudinal’ profile of glycaemic control over the past 2 to 3 months [6]. In assessing if a patient has appropriate glycaemic control, a simple and convenient expedient involves categorisation of HbA1c measurements as ‘satisfactory’ or ‘unsatisfactory’ according to a predetermined cut-off value [7]. Our endocrine paediatricians currently use an *a priori* cut-off HbA1c level of ≤9% (≤75 mmol/mol) as an indicator of satisfactory glycaemic control, although trends in the HbA1c data are also considered along with an individual’s clinical circumstances. Due to the complex nature of the endocrine system and its pathology, and the sources of variability that influence glucose control we hypothesized that multivariate mixed-effects, logistic regression modelling of routinely recorded clinical data would provide a potentially useful means of identifying patient characteristics which influence glycaemic control. Logistic regression modelling is a commonly used method of analysis for estimating the probability (specifically via the Odds Ratio [OR]), of a variable on categorical response data [8]. Such categorical data are frequently ‘information poor’ such that in the present study the recorded response (dependent variable) can be 1 of only 2 possibilities (‘satisfactory’ or ‘unsatisfactory’ glycaemic control). The specific aim of this study was to apply such an approach to screen and quantify characteristics recorded routinely in the paediatric endocrine clinic in order to develop a predictive population model which could be applied to improve glycaemic control in young children and adolescents with T1DM.

## Methods

### Patients and data handling

Data were extracted from a computerised database held by the Queensland Diabetes Centre, Mater Health Services, Brisbane, Queensland, Australia. Data from paediatric and adolescent patients between 1 and 19 years of age who visited the Queensland Diabetes Centre between January 1^st^ 1998 and January 1^st^ 2001 were included in the analysis. Prior written approval to access the database was obtained from the ethics committees of Mater Health Services, and The University of Queensland. The data were anonymized upon retrieval from the database. All data underlying this research has been included as supplementary material (S1–S3 Tables). The dataset was stratified into a training (70%) and validation (30%) dataset. The training dataset was used for the model development.

Information was obtained on the following patient characteristics: age, disease duration, fractional disease duration (disease duration/age), age at diagnosis of T1DM, baseline HbA1c, height, weight, body mass index (BMI), insulin dose per injection, total daily insulin dose, visit interval to the hospital. Categorical variables were sex, and consultation(s) with a diabetes educator, dietitian, or psychologist over the previous 3 months. Each visit was stratified with respect to unsatisfactory/satisfactory glycaemic control, and differences at the start of the observation period between the two groups were tested using an independent two-sample t-test for the continuous variables [9]. The baseline HbA1c level was the measurement obtained at the first visit after 1^st^ January 1998 for an individual patient. Patients who moved to Brisbane at a later stage or changed hospitals during the observation period had fewer observations over the study period. For modelling purposes, and for easier interpretation of the OR estimates, the fractional disease duration variable was coded as ‘disease duration/age**·**10’, so that an increase of 1 is equivalent to an increase of 0.10 in fractional disease duration. Recent changes in the guidelines for reporting HbA1c measurements are being implemented in Australia, in order to standardize the reporting of HbA1c concentration in SI units (mmol/mol) which are now used worldwide [10]. However, no conversion was made from the original collected data, therefore all HbA1c concentrations are reported here as percentages, but this scaling does not affect the subsequent analysis in any way. The cut-off HbA1c value of 9% used here is equivalent to an HbA1c concentration of 75 mmol/mol [11].

### Missing data

Missing values for height and weight not recorded during a consultation were replaced with the observation at a previous or a future recorded visit of the same patient, with the timely closest value chosen to replace the missing value. The corresponding BMI values of those observations were calculated with the new weight and/or height values. If multiple consecutive height or weight observations were missing they were replaced by median height values obtained from the World Health Organization Child Growth Standards corresponding with a patient’s age [12], or by median weight values reported by the Centers for Disease Control and Prevention for children older than 12 years [13]. Observations were not included in the analysis when an observation of a different variable was lacking. The visit interval between a previous visit and the first visit of the observation period was unknown, therefore this was changed to the mean visit interval of 3.5 months.

### HbA1c analysis

HbA1c levels were measured using a latex immunoagglutination inhibition assay on a DCA 2000 analyser (Bayer Diagnostics, Sydney, NSW, Australia). The validated working range for this method ranged from 2.5% to 14.0% for HbA1c. The coefficient of variation was 2.0%– 3.4%.

### Logistic regression model building

Logistic regression is commonly used to estimate the probability and the OR of a stimulus or condition on a dichotomous response. The resulting probability estimated by the model is the probability of the outcome being equal to 1, which in the present study represented ‘satisfactory’ glycaemic control. The stimulus represents a patient characteristic that could potentially influence the dependent variable, which presently was the achievement of satisfactory glycaemic control (HbA1c <9%). A mixed-effects population modelling approach using a random effects model with variability on the intercept term was used to identify the ‘typical’ population response, but which also preserves to an extent the ‘individuality’ of a patient’s response [14]. A random-effects model was chosen for data analysis to increase the understanding of the underlying mechanism of individual risk factor on HbA1c [15]. The multivariable logistic regression modelling strategy presently used is described more fully elsewhere [16]. Several assumptions were tested to ensure that model outcomes were reliable. Multicollinearity was tested using a variance inflation factor (VIF) metric to ensure that two or more explanatory variables included in a multiple logistic regression model were not highly correlated; otherwise this could result in a change of the regression coefficients of all affected variables, and correct interpretation of the OR would be impossible as the outcome would not be solely influenced by a single variable. If two patient characteristics showed high correlation (VIF >3), the least significant variable was excluded from the model. The model was developed using a stepwise logistic regression approach involving forward inclusion and backward elimination [16]. Inclusion of a variable was deemed to be statistically significant at p <0.05. At each step, variables were included for testing if deemed to be clinically or biologically plausible. The OR was calculated by taking the natural exponent of the regression coefficients (β) estimated by the model. Model building was continued until the addition of a new variable did not result in further statistically significant improvement of the model. The significant improvement of the model was judged by the objective function value (OFV) which is a goodness-of-fit metric generated internally during fitting of a model to the data, and is equal to -2 **·** log likelihood value, which approximates a *X*^*2*^ distribution [17]. The addition of a variable was considered to have made a significant improvement to the fit of the model when the OFV decreased by at least 3.84 with 1 additional degree of freedom [9].

### Model evaluation and validation

The sensitivity and the specificity of the developed model were determined using a receiver operating characteristic (ROC) curve [18]. The ROC curve gives an insight into the predictive value of the model in terms of its ability to correctly describe patients having/not having satisfactory glycaemic control. The balance between high sensitivity and high specificity depends on the goal of the study; presently we aimed to predict those patients having satisfactory control while minimizing misspecifications of the model. Consequently, the predictive ability of the model was chosen at the middle of the arch of the ROC curve. The 95% confidence interval on the ROC curve was generated using a 20,000 sample bootstrap. Internal validation was performed using a nonparametric bootstrap analysis (with replacement) to assess the robustness and stability of a candidate model, in which the model was fitted to 1000 randomly selected subsets of the main data set [19]. When the 95% (2.5^th^– 97.5^th^) percentile confidence interval (CI) of the OR included unity (i.e. no difference in OR with an increase of 1 unit), the variable was discarded since its effect on the outcome would not be significant after bootstrap analysis. The predictive abilities of the model were checked by estimating the probabilities of satisfactory control on the validation dataset, while incorporating only the fixed effects of the final model.

### Software

Modelling was performed using NONMEM version 7.3 [17] in conjunction with Perl-speaks-NONMEM (PsN) version 4.4.8 [20]. Data assembly and plots were generated using R version 3.3.2 in combination with RStudio version 0.99.887 [21–23]. ROC curves were created using the pROC package in R [24]. Multicollinearity between patient characteristics was examined using the IBM SPSS Statistics 23 package [25].

## Results

### Patients and data

**Fig 1:**
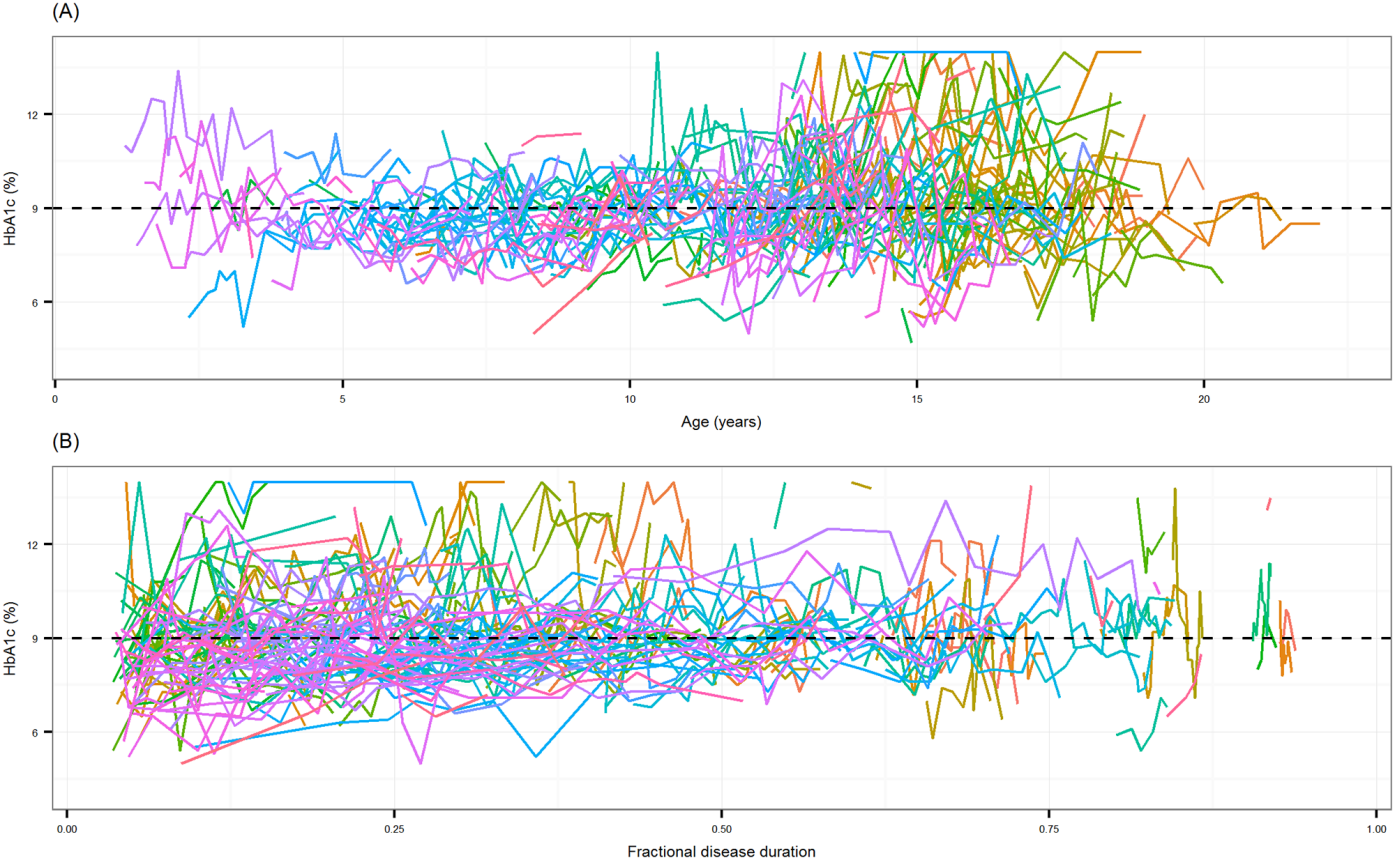
**A: HbA1c measurements (%) versus age (years). B: HbA1c measurements (%) versus fractional disease duration.** Individual lines connect all observations for a patient. Dashed lines show the HbA1c cut-off value (9%) for satisfactory glycaemic control.

The data comprised 1852 HbA1c measurements in 288 patients (153 female, 135 male) with T1DM, aged 1.2 to 19.8 years old at the start of the observation period. Fig 1A and 1B show individual HbA1c measurements versus age, and fractional disease duration during the study period, respectively; observations for each patient were connected to illustrate a progression trend. The dashed lines indicate the cut-off value for HbA1c (9%) that was used for categorisation. There were a total of 897 ‘satisfactory’, and 955 ‘unsatisfactory’ HbA1c observations during the study period. The characteristics of the patients at the beginning of the observation period are presented in Table 1, in which the mean baseline HbA1c (HbA1c level (%) at the beginning of the observation period) was 7.8% for patients having satisfactory glycaemic control, and 10.3% for patients having unsatisfactory control. The first clinic visit during the observation period was on average 3.4 years after T1DM diagnosis. In both groups, patients visited a healthcare professional in addition to an endocrinologist in 2 out of 3 hospital visits. All patients were receiving a conventional dosage regimen of 1 to 4 subcutaneous insulin injections per day. A self-titrated combination of short- and intermediate-acting insulin was the most common type of treatment for 259 patients (89.9%), while 11 patients (3.8%) had premixed insulin, 8 patients (2.8%) had a combination of short-acting and long-acting insulins, 7 patients (2.4%) had intermediate-acting insulin, 2 patients (0.7%) had a combination of short-acting, intermediate-acting and long-acting insulin, and 1 patient (0.35%) was receiving both short-acting and premixed insulin. The training and validation dataset had similar patient characteristics, and summary statistics have been included as supplementary material (S4 Table).

**Table 1:** Patient characteristics at the start of the observation period.

Patient characteristics	Satisfactory glycaemic control (HbA1c < 9%)	Unsatisfactory glycaemic control (HbA1c ≥ 9%)	Overall range
Total number of patients	288		-
Number of patients	169	119	
Total number of observations ^a^	897	955	-
Sex—Female	85	68	-
Sex—Male	84	51	
Age (years)*	10.9 (4.39)	11.9 (4.13)	1.2–19.8
Age at diagnosis (years)	8.1 (3.95)	7.5 (4.03)	0.7–16.5
Disease duration (years)**	2.8 (3.17)	4.4 (3.80)	0.08–16.2
Fractional disease duration **	0.24 (0.23)	0.36 (0.26)	0.01–0.93
Weight (kg)*	42.7 (18.53)	47.8 (19.65)	8.2–98.5
Height (cm)	143.6 (23.94)	148.2 (23.61)	72.5–192.6
BMI (kg/m^2^)*	19.5 (3.52)	20.6 (4.03)	11.7–35.7
HbA1c (%)**	7.8 (0.90)	10.3 (1.30)	5.0–14.0
Insulin dose per injection (U)**	15.9 (10.66)	20.8 (13.26)	1.5–68.5
Daily insulin dose (U)**	35.8 (25.35)	50.2 (30.85)	2.0–137
Visit Interval (months) ^a^	3.5 (2.33)		0.12–27.8
Diabetes educator^b^	98	71	-
Dietitian^b^	68	40	
Psychologist^b^	54	32	

Mean (sd). Fractional disease duration = disease duration/age, visit interval = time interval between two visits,

^a^ = during full observation period,

^b^ = number of patients that had a visit to a healthcare professional within the last 3 months before start of observation period,

* = *p*-value < 0.05,

** = *p*-value <0.001

### Model building

An overall probability of 51% of achieving satisfactory glycaemic control was estimated using a logistic model including only the intercept term, prior to the inclusion of candidate patient characteristics. The estimated variance (ω^2^) on the intercept term was estimated as 4.39. The results of the stepwise model building are shown in Table 2. A total of 3 forward selection steps were required to develop the final model. Inclusion of baseline HbA1c resulted in a decrease of 124 in the OFV, and this variable was retained in the model at the first step as the most significant patient characteristic for glycaemic control. The inclusion of age also produced a significant decrease in the OFV (-19.39). Fractional disease duration (representing the proportion of life with diagnosed T1DM) was added as the final significant characteristic producing a significant decrease of -11.78 in the OFV. Age at which T1DM was diagnosed and disease duration also were significant, but both factors showed marked multicollinearity with fractional disease duration, therefore they were subsequently excluded from the model building. No interaction terms were retained in the final model. Backward elimination did not necessitate the removal of any variable from the model. The ω^2^ on the intercept term was reduced from 4.39 to 2.93 after inclusion of all significant variables. Corresponding OR and 95%-CI estimates for the final model after bootstrap analysis are shown in Table 3. The baseline HbA1c gave an OR of 0.30 for every 1% increase of the HbA1c measurement at the beginning of the observation period. Age of the patient at the time of HbA1c measurement resulted in an OR of 0.88 for every 1 year increase in age, while fractional disease duration had an OR of 0.80 per 0.10 increase in fractional disease duration.

**Table 2:** Stepwise model building results.

Step	Patient characteristic	OFV	ΔOFV	Δdf
0	Intercept	1424.52	NA	NA
1	Baseline HbA1c	1300.47	-124.05**	1
2	Age	1281.08	-19.39**	1
3	Fractional disease duration	1269.30	-11.78**	1

OFV = Objective Function Value, ΔOFV = Difference in OFV between two competing models, Δdf = degrees of freedom between two competing models, NA = Not applicable,

** = *p*-value < 0.001.

**Table 3:** Parameter estimates, regression coefficients and odds ratios with the 95% CI of the final multivariable logistic regression model and bootstrap results (n = 1000).

	Model estimates		Bootstrap results		
Patient characteristic	β (RSE%)	OR	Median β (RSE%)	Median OR	OR 95% CI
Intercept	12.7 (12.9%)	-	12.72 (12.98%)	-	-
Baseline HbA1c (%)	- 1.2 (13.7%)	0.30	-1.19 (13.75%)	0.30	0.20–0.41
Age (years)	- 0.13 (31%)	0.88	-0.13 (31.2%)	0.88	0.80–0.94
Fractional disease duration	- 0.22 (36.2%)	0.80^a^	-0.22 (36.13%)	0.80^a^	0.66–0.93

β = regression coefficient, RSE% = relative standard error in %, OR = Odds Ratio, OR 95% CI = 95% confidence interval of the odds ratio,

^a^ = for every 0.10 increase in fractional disease duration

### Model evaluation and validation

**Fig 2:**
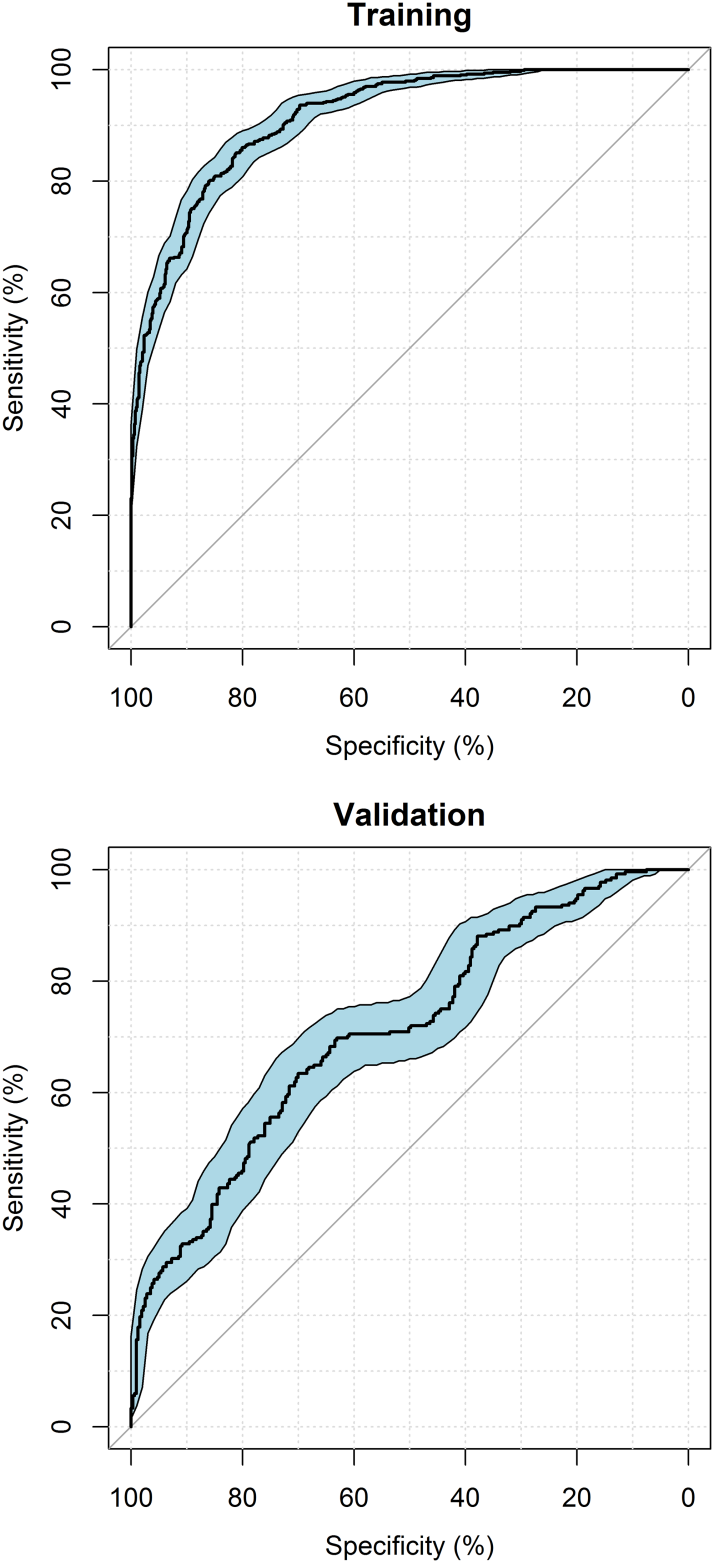
Receiver operating characteristic curves of the training (AUC = 91.5%) and validation (AUC = 71%) models. The curved black line shows the sensitivity versus specificity of the multivariable logistic regression models. The diagonal lines describe the random line for a model with no predictive abilities. The blue shaded area represents the 95% confidence interval.

The parameters included in the final model were tested for multicollinearity. VIFs were all less than 3 indicating that there was low collinearity between variables. The ROC curves of both the training and validation datasets (Fig 2) showed that this model was able to describe the data in both datasets. The area-under-the-curve (AUC) of the training dataset was 91.5% whereas the validation dataset had an AUC of 71%. The lower AUC is the result of the simulated probabilities for satisfactory glycaemic control while only including the fixed effects. The results of the bootstrap (Table 3) showed that the median values of the parameter estimates agreed closely with the corresponding values of the final model. None of the 95% CIs for an OR estimated in the final model included 1, indicating that all included variables provided a significant improvement in the fit of the model to the data.

## Discussion

The outcome of the mixed-effects logistic regression modelling clearly showed that glycaemic control in the early stages of disease was the best explanatory variable of subsequent control in young children and adolescents with T1DM; unsatisfactory control predicted poor future control. Furthermore, age and fractional disease duration also were significant predictors of glycaemic control. The final model predicted that patients having elevated HbA1c levels had a higher probability of future unsatisfactory HbA1c levels. In support, previous research showed that HbA1c at the commencement of a study period was a predictor of future glycaemic control, and that those patients with unsatisfactory glycaemic control had higher intra-individual variability between HbA1c monitoring events [26]. Others reported that poor metabolic control in the second year after diagnosis was the best predictor for the HbA1c between 3 and 6 years after diagnosis. [27]. The results of other studies supported our findings in that HbA1c levels as well as age are significant predictors of future glycaemic control [28,29].

The present study showed that a decrease in satisfactory glycaemic control and increased variability in control in older children may partly be due to factors associated with the onset of puberty. Fig 1A showed a large increase in variability of HbA1c levels at about 13 years of age. Indeed, it was previously reported that decreased glycaemic control often occurs at the onset of puberty [30,31], which may be the result of complex changes in physiology, and the influence of psychosocial factors at this age. Besides dietary changes during puberty there are surges in human growth hormone [32], which is an insulin antagonist [33]. Decreased compliance and frequency of blood glucose monitoring (perhaps as a reaction to perceived peer pressure), and increased levels of stress also may affect glycaemic control in adolescents [4,34].

Two significant variables for glycaemic control were closely related; fractional disease duration and disease duration, with fractional disease duration being identified as the stronger predictor for glycaemic control. Fractional disease duration incorporates aspects of disease duration and age of diagnosis in a single variable which is an advantage compared to implementing the variables on their own. As an example, a 15-year-old patient with a disease duration of 5 years would have a fractional disease duration of 0.33, compared to a 6-year-old patient who has been living with diabetes for 5 years where the fractional disease duration is 0.83; those living with diabetes for a longer proportion of their life are at higher risk of having unsatisfactory HbA1c levels. Although some studies found that age at T1DM diagnosis was a positive predictor for unsatisfactory glycaemic control [35,36], this could not be included in the final model due to the multicollinearity issues as described above.

The mean HbA1c value of 9.16% in our patients was slightly higher than recorded for some other studies in type 1 diabetic children [37–39], which could simply be a reflection of the fact that many of our patients had been referred to the clinic because of significant difficulties with their management elsewhere such as in regional areas. Consultations with healthcare professionals other than an endocrinologist did not predict a significant improvement in glycaemic control. The fact that many patients had regular appointments with at least one healthcare professional regardless of their current HbA1c status may partly explain why this variable did not achieve the level of significance necessary for inclusion in the final model. Nonetheless, there is a consensus that regular consultations with healthcare professionals to continuously educate patients about the dosing and administration of insulin, the monitoring of blood sugar levels, and counselling on aspects of lifestyle changes remain important for achieving satisfactory glycaemic control [40,41]. However, the overall effect of diabetes education for children and their families remains equivocal [42,43], and further research on methods of education and support in this population is required to improve the chances of better glycaemic control.

The final model resulted in an AUC of 91.5% under the ROC curve of the training dataset and an AUC of 71% for the validation dataset, suggesting that there are other unidentified factors which also are predictors for glycaemic control in these children. This also may be a consequence of the amount of data available in the present study, especially since binary categorical data is relatively ‘information-poor’, compared to continuous data. Thus, the discriminatory power of such an analysis could be augmented with increased amounts of data. Therefore, data collection needs to be extended to other centers both in Australia and elsewhere. The data used in the present modelling have been provided which may facilitate an external validation of the identified predictive patient characteristics in the study. The final model was able to identify patients with satisfactory/unsatisfactory control in approximately 85% of cases using the 3 most significant factors which are routinely documented in T1DM.

There are multiple hazards and pitfalls associated with multivariable logistic regression modelling, and while this approach is often used in biomedical research many published studies have not embraced the rigor needed to correctly asses the value, stability, or significance of the reported models [44,45]. Presently, our paediatric patients with T1DM are a ‘difficult’ cohort of patients in which there is considerable variability, together with the risk of confounding variables and multicollinearity. In view of the rapid increase in the prevalence of diabetes in children and adults [46], this study is timely and relevant and illustrates how a multivariable logistic regression modelling approach has much potential for application in the therapeutics and epidemiology of type 1 diabetes. It was recognised from the outset that the format of the data had to be categorical in nature, because the outcome of direct interest to our endocrinologists was whether a given patient’s glycaemic control fell above or below an *a priori* HbA1c threshold. In future studies it may be of interest to also model more information-rich data using the actual HbA1c level as a continuous dependent variable.

In conclusion, the present study identified and quantified the influence of several factors in a mixed-effects (population) logistic regression model that has the potential to assist clinicians to identify paediatric patients who are at increased risk of having unsatisfactory glycaemic control, and who would require more extensive monitoring, support, or targeted interventions.

## Supporting information

S1 Table Anonymised dataset with patient characteristics. (CSV)

S2 Table Anonymised dataset with HbA1c observations and fractional disease duration. (CSV)

S3 Table Anonymised NONMEM dataset. (CSV)

S4 Table Patient characteristics of training and validation dataset. (DOCX)
