# Supplementary material for: Prediction of glycaemic control in young children and adolescents with type 1 diabetes mellitus using mixed-effects logistic regression modelling
Source: PLoS One. 2017 Aug 2;12(8):e0182181. doi: 10.1371/journal.pone.0182181 (PMC5540397; doi:10.1371/journal.pone.0182181)
Supplement: S4 Table — (DOCX) [file pone.0182181.s004.docx]

**S4. Patient characteristics at the start of the observation period for the training and validation dataset.**

|  | **Training dataset** | | | **Validation dataset** | | |
| --- | --- | --- | --- | --- | --- | --- |
| **Patient characteristics** | **Satisfactory glycaemic control  (HbA1c < 9%)** | **Unsatisfactory glycaemic control  (HbA1c ≥ 9%)** | **Overall range** | **Satisfactory glycaemic control  (HbA1c < 9%)** | **Unsatisfactory glycaemic control  (HbA1c ≥ 9%)** | **Overall range** |
| Total number of patients | 202 | | - | 86 | | - |
| Number of patients | 123 | 79 | - | 46 | 40 | - |
| Total number of observations^a^ | 629 | 638 | - | 268 | 317 | - |
| Baseline HbA1C (%) | 7.79 (0.88) | 10.34 (1.29) | 5-14 | 7.68 (0.95) | 10.33 (1.34) | 5.4 – 14 |
| Age (years) | 10.65 (4.51) | 11.68 (4.11) | 1.2 – 19.84 | 11.47 (4.03) | 12.30 (4.18) | 1.55 – 19.39 |
| Fractional disease duration | 0.251 (0.24) | 0.37 (0.26) | 0.01 – 0.93 | 0.21 (0.20) | 0.33 (0.26) | 0.005-0.93 |
| Visit Interval (months) ^a^ | 3.5 (2.45) | | 0.12 – 27.8 | 3.39 (2.06) | | 0.48-15 |

Mean (sd). Fractional disease duration = disease duration/age, visit interval = time interval between two visits, a = during full observation period
